# Coarse-Grained Model of the Sodium Dodecyl Sulfate Anionic Surfactant Based on the MDPD–Martini Force Field

**DOI:** 10.1021/acs.langmuir.5c05313

**Published:** 2026-03-04

**Authors:** Luís H. Carnevale, Gabriela Niechwiadowicz, Panagiotis E. Theodorakis

**Affiliations:** 86906 Institute of Physics, Polish Academy of Sciences, Al. Lotników 32/46, 02-668 Warsaw, Poland

## Abstract

The sodium dodecyl sulfate (SDS) surfactant is widely used in various applications, such as household products (e.g., shampoos, toothpaste, detergents, and cleaning products) and food manufacturing (e.g., emulsifiers). To investigate its properties via computer simulation, various models have been developed, including coarse-grained (CG) models that are suitable for capturing a surfactant’s self-assembly and fundamental properties for aqueous systems with a surfactant, such as surface tension. Here, we present a CG model for SDS/water systems for many-body dissipative particle dynamics (MDPD), which is based on the MDPD–Martini force field (FF). In the model, charged groups, namely, the SDS sulfate headgroup and the sodium cation, are explicitly modeled following the standard mapping of the Martini force field for molecular dynamics (MD), while the remaining interactions have been obtained from previous MDPD–Martini models for lipid systems, thus demonstrating their transferability. Various relevant system properties, such as the coherent scattered intensity and surfactant distribution at the liquid–vapor surface, are investigated, and results are compared to those obtained by MD simulations and experiments at different surfactant concentrations. Our findings indicate that MDPD–Martini models can offer a credible alternative to MD–Martini models for systems with explicit charges as shown here for SDS. Moreover, MDPD–Martini models reproduce nicely the experimental surface tension isotherm, in contrast to MD simulations. In view of the transferability of the MDPD–Martini interactions, the model parameters of this study can be tested and used to simulate a wider range of soft-matter systems.

## Graphical abstract

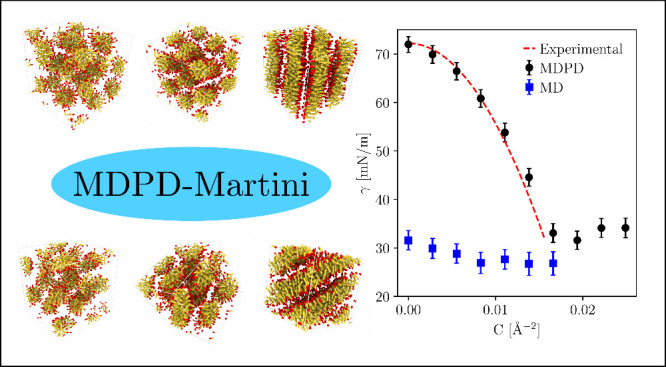

## Introduction

Surfactants are commonly used in various applications as detergents,^1^ emulsifiers,^2,3^ wetting and foaming agents,^4,5^ etc. The broad use of surfactants in applications is due to their amphiphilic nature; they consist of a hydrophilic part and a hydrophobic part. For this reason, they can favorably adsorb at interfaces, where they can reduce the surface tension,^6^ thus alleviating the tension between different phases. Moreover, at concentrations above a critical aggregation concentration (CAC), they can form aggregates of different size and shape, for example, spherical micelles, etc.;^4^ therefore, various properties of aqueous systems with a surfactant strongly depend on surfactant concentration. Moreover, surfactants can be nonionic, such as alkyl ethers,^7–9^ ionic, including both anionic^10,11^ and cationic^12^ surfactants, and zwitterionic, including both positively and negatively charged groups.^13^

Sodium dodecyl sulfate (SDS) is an anionic surfactant that is widely used in industry. For example, it is commonly used in commercial products, such as shampoos and cleansers. For this reason, understanding its properties has been the focus of both experimental and theoretical research alike, in particular properties such as the size and shape of SDS micelles and determining the CAC above which these occur.^14–26^ At the same time, molecular simulation has been an important tool for investigating aqueous systems with SDS since it can often provide insights beyond experimental capabilities by tracking each molecule at all times during the simulation. Still, simulating systems with a surfactant is challenging even for molecular dynamics (MD) simulations of coarse-grained (CG) models, since the time scale involved to fully capture certain phenomena, such as the self-assembly and diffusion of surfactant aggregates, would require adequately long simulations or following simulation protocols (e.g., simulation annealing methods^27^) for efficient sampling, for example, to overcome metastable energy minima toward a global minimum. Despite previous MD studies, which have provided valuable insights into the properties of water/SDS systems,^28–35^ many challenges persist, such as reliable surface tension measurements. For this reason, there has been a quest to improve CG models, including further reduction of the associated computational costs of these methods. In this regard, dissipative particle dynamics (DPD)^36–38^ and many-body dissipative particle dynamics (MDPD)^39–43^ simulation methods that rely on soft, short-range interactions have emerged as a suitable alternative to MD simulation models that usually rely on hard-core interactions (e.g., Lennard-Jones). Moreover, the sodium ion has thus far mostly been part of the hydrophilic head of the SDS surfactant, thus represented by an uncharged interaction site in MDPD models.^40–42^ However, it has recently been shown that sodium ions can actually be represented as separate point charges^39^ as in various all-atom and CG models commonly employed in MD simulations (e.g., Martini^44,45^ ), instead of using a smeared charge approach due to the soft nature of the potential in DPD and MDPD models.^36–38^ Furthermore, avoiding the so-called charge collapse related to the presence of long-range interactions due to the presence of point charges in the simulations has opened new possibilities for developing MDPD force fields that rely on point charges as separate beads,^39^ a feature that is indispensable for developing a general-purpose force field (FF). Hence, this specific aspect is key for the MDPD–Martini models,^46,47^ which will be presented here for aqueous systems with the SDS surfactant.

The MDPD–Martini FF^46,47^ is generally based on the Martini “LEGO” approach and its mapping^44,48–50^ for describing the interactions between the different bead types. It has been used for different lipid systems offering a significant acceleration of simulations with respect to those relying on MD–Martini models without compromising the quality of results regarding various relevant properties. Furthermore, the self-assembly process of lipid bilayers was shown to be 4–7 times less computationally expensive, depending on the system size, when comparing MDPD–Martini with MD–Martini.^46,47^ This is a significant advantage with respect to MD–Martini simulations, thus offering in general the capability of simulating larger systems for longer times. Moreover, MDPD models can reproduce hydrodynamic interactions, which is generally challenging for MD simulations, and they can reach equilibrium states of soft-matter complex systems more easily than in the case of MD simulations.^51^ Here, we proceed one step further and show how the MPDD–Martini models apply in the case of water/SDS systems with the sodium particles being represented by individual point charges following the mapping recipe of MD–Martini models.^48,52^ A key feature of the MDPD–Martini approach is the transferability of the interactions (“LEGO approach”), which renders this FF suitable for simulating a range of different systems (general-purpose), as in the case of the standard MD Martini. For this reason, MD–Martini models have been applied for a wide range of systems, such as proteins,^53–55^ polymers,^56^ carbohydrates,^57^ glycolipids,^58^ glycans,^59^ DNA,^60^ RNA,^61^ water,^62^ and various solvents.^63^ Moreover, various extensions include simulations for specific pH values,^64^ chemical reactions (reactive Martini),^65^ and the Go̅Martini approach for proteins,^66^ which has also been integrated into the Martini 3.0 release.^52^

In this study, we carried out MDPD simulations based on the MDPD–Martini FF for water/SDS systems for various surfactant concentrations. Several properties for these systems have been calculated and juxtaposed with previous MD–Martini results and experiments.^28,33,67,68^ The MDPD–Martini interactions previously obtained for lipid systems^46,47^ demonstrate transferability to the current water/SDS system. Moreover, the sodium cation, with its first hydration shell, and SDS anionic headgroups are represented by distinct Q_d_ and Q_a_ Martini-type beads, respectively. We find that the MDPD–Martini models perform better for properties such as the surface tension isotherm in comparison with the MD–Martini model and in good agreement with the experiment.^67^ Moreover, the self-assembly morphologies as a function of concentration agree well between the MD and MDPD models, as well as other properties, such as the density distribution of the surfactant at the liquid–vapor surface and its thickness and the coherent scattered intensity. Thus, while our study provides the MDPD–Martini FF parameters for SDS/water systems with explicit charges, it also opens possibilities for simulating a wider range of systems due to the transferability of the currently developed MDPD–Martini FF interaction matrix, which can be further tested in practice in future work.

## Materials and Methods

### Molecular Dynamics

Simulations for the MD and the MDPD models are carried out by using LAMMPS.^69,70^ Following the usual Martini approach, MD simulations were run with a shifted Lennard–Jones potential where the energy goes to zero smoothly between an inner and an outer cutoff (*r*_in_ = 0.9 nm and *r*_out_ = 1.2 nm, respectively). Long-range electrostatic interactions were computed with the particle–particle particle–mesh (pppm) method and by setting the electric permittivity to 80 following previous studies.^33,71^ This method takes into account the electrostatic interactions explicitly through the Coulomb potential for particles up to the computational cutoff, and the longer-range interactions are computed from the Ewald sum on reciprocal space. The temperature and pressure in the simulations were controlled through the Nosé–Hoover thermostat as implemented in LAMMPS. The temperature was kept at 300 K, and the pressure at 1 atm. A 20 fs time step was used in all MD simulations.

The Martini model for the SDS surfactant is illustrated in Figure 1. It consists of the Q_a_ headgroup bead with a negative electric charge and three C_1_ tail beads tethered together with harmonic interactions to form the SDS chains. An equal number of sodium cations to the total number of SDS chains is also included in the simulation box to neutralize the system. These cations are represented by the Q_d_ bead type. All simulations were performed with water as an explicit solvent modeled by P_4_ Martini beads. A nonpolarizable water model was used due to its much lower computational cost when compared to that of the Martini polarizable water model. Furthermore, most models in the literature use nonpolarizable water for the SDS/water system, making it a suitable option to validate our MD simulations.

**Figure 1:**
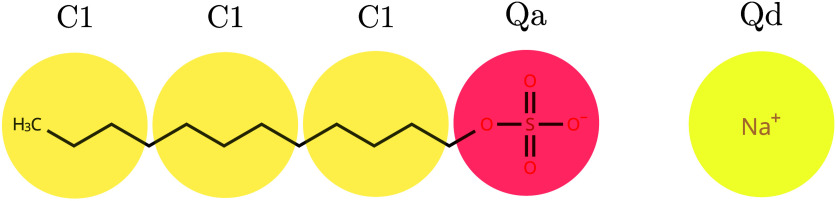
Coarse-grained representation of the SDS surfactant and sodium counterion used in both MD and MDPD simulations. SDS beads are tethered together with harmonic interactions (see the text for details), while each sodium ion, with its first hydration shell, is represented by a point charge bead, which is not bonded to the SDS chain.

The specific Lennard-Jones interaction parameters, namely, ϵ_*ij*_ and σ*_ij_*, for each pair of beads are reported in Table 1. The harmonic potential between bonded beads in a surfactant molecule was set to have a force constant (*K*) of 1250 kJ mol^–1^ nm^–2^ and a bond length (*r*_0_) of 0.47 nm. A harmonic cosine potential among three consecutive bonded beads was also used with a force constant (*K*) of 25 kJ mol^–1^ and an equilibrium angle (θ_0_) of 180°.^33,48^

**Table 1:** Lennard-Jones *ϵ_ij_* Parameter for Each Bead Interaction in Kilojoules per Mole^a^

	P_4_	Q_d_	Q_a_	C_1_
P_4_	5.0	5.6	5.6	2.0
Q_d_		5.0	5.6	2.0
Q_a_			5.0	2.0
C_1_				3.5

a *i* and *j* represent different bead types. The distance, *σ_ij_*, was set to 0.47 nm, except for the Q_d_–C_1_ and Q_a_–C_1_ interactions, where it was set to 0.62 nm.^33^.

### Many-Body Dissipative Particle Dynamics

The MDPD method has been applied to investigate a range of different systems, which includes different types of fluids^51,72–77^ and their properties, such as surface tension.^78^ However, it is also suitable for investigating multiphase and multicomponent soft-matter systems, such as systems with surfactants.^39,42^ During a standard MDPD simulation, the Langevin equation of motion is solved for each particle of the system as in MD,^79^ but the pairwise forces are a direct input in the case of MDPD simulations. The Langevin equation reads

1 $$m\frac{dv_{i}}{dt}=\underset{j\neq i}{\sum }F_{ij}^{C}+F_{ij}^{R}+F_{ij}^{D}$$

where **F** ^C^ is the conservative force on each particle and **F** ^R^ and **F** ^D^ are the random and dissipative forces, respectively, which act as a thermostat. Hence, the latter forces are related through the fluctuation–dissipation theorem. The mass, *m*, of the beads is the same for all particles and set to unity.

A main difference between the MDPD method and its predecessor, DPD,^80–85^ lies with the expression for the conservative force between particles, which includes attractive interactions and is derived from density-dependent potentials. The most common form of the conservative force reads^73^

2 $$F_{ij}^{C}=A\omega^{C}\left(r_{ij}\right)e_{ij}+B\left(\overset{®}{\rho_{i}\;}+\overset{®}{\rho_{j}\;}\right)\omega^{d}\left(r_{ij}\right)e_{ij}$$

where *A* < 0 and *B* > 0 are the parameter strengths of the attractive and repulsive parts of the potential, respectively. Here, *r*_*ij*_ indicates the distance between particles, while **e**_*ij*_ is the unit vector from particle *i* to *j. ω* ^C^(*r*_*ij*_) and ω^d^(*r*_*ij*_) are linear weight functions, namely

3 $$\omega^{C}\left(r_{ij}\right)=\{\begin{array}{cc} 1-\frac{r_{ij}}{r_{c}}, & r_{ij}\leq r_{c}\\ 0, & r_{ij}>r_{c} \end{array}$$

where *r*_c_ is a cutoff for the interactions, usually set to unity. Also, ω^d^(*r*_*ij*_) is defined by the same expressions as ω^C^(*r*_*ij*_), but interactions have a smaller value than *r*_c_, namely, *r*_d_ = 0.75.

The repulsive term of the potential depends on the local neighborhood densities, ρ̅_*i*_ and ρ̅_*j*_, and are given as follows:

4 $${\mathrm{\rho ̅}}_{i,j}=\underset{0<r_{ij}\leq r_{d}}{\sum }\frac{15}{2\pi {r_{d}}^{3}}{\left(1-\frac{r_{ij}}{r_{d}}\right)}^{2}$$

while, the random and dissipative forces are

5 $$F_{ij}^{D}=-\sigma \omega^{D}\left(r_{ij}\right)\left(e_{ij}\cdot v_{ij}\right)e_{ij}$$

6 $$F_{ij}^{R}=\xi \omega^{R}\left(r_{ij}\right)\theta_{ij}\Delta t^{-1/2}e_{ij}$$

where σ is the dissipative strength, ξ the strength of the random force, **v**_*ij*_ the relative velocity between particles, and θ_*ij*_ a random variable from a Gaussian distribution with unit variance. The fluctuation–dissipation theorem dictates that σ and ξ be related by the following expression

7 $$\sigma =\frac{\xi^{2}}{2k_{B}T}$$

The weight functions for the random and dissipative forces are

8 $$\omega^{D}\left(r_{ij}\right)={\left[\omega^{R}\left(r_{ij}\right)\right]}^{2}=\{\begin{array}{cc} {\left(1-\frac{r_{ij}}{r_{c}}\right)}^{2}, & r_{ij}\leq r_{c}\\ 0, & r_{ij}>r_{c} \end{array}$$

Finally, the system’s temperature has been kept constant throughout the simulations and set to unity (MDPD units). All simulations were done with a time step (*Δt*) of 0.01 and dissipative strength (σ) of 4.5.

The interaction levels and their respective *A*_*ij*_ parameters for the nonbonded attractive interactions between particles and ions are presented in Table 2 and have been obtained from our previous work with lipid membranes where parametrization was done based on water–octanol partitioning coefficients.^46,47^ The bead labels have been renamed to follow the Martini convention for ease of comparison. Here, these interactions are applied for the study of the aqueous systems laden with the SDS surfactant and by setting Q_d_ interactions equal to Q_a_, while *B* = 25 remains constant due to the “no-go theorem” in MDPD.^86^ The types of beads and the groups they represent are illustrated in Figure 1 and are the same for the MD and MDPD models following Martini mapping.

**Table 2:** MDPD–Martini FF Interaction Matrix for Parametrized MDPD Beads Organized in Six Interaction Levels (I–VI) with the Corresponding Attractive Parameters^a^

	P_4_	Q_0_	Q_a_	N_a_	C_1_	C_3_
P_4_	I	I	I	II	VI	V
Q_0_	I	IV	IV	II	VI	V
Q_a_	I	IV	IV	II	VI	V
N_a_	II	II	II	III	VI	V
C_1_	VI	VI	VI	VI	VI	VI
C_3_	V	V	V	V	VI	VI

a *A*_*ij*_ values of −50 (I), −43 (II), −34 (III), −30 (IV), −28 (V), and −26 (VI). These values were obtained by keeping the repulsive parameter constant for all interactions at *B* = 25.

To build the SDS molecules, harmonic bond and angle interactions are used

9 $$E_{\mathrm{bond}}=\frac{k}{2}{\left(r_{ij}-r_{0}\right)}^{2}$$

for the bonds when *k* = 150 and *r*_0_ = 0.5 and

10 $$E_{\mathrm{angle}}=\frac{k_{A}}{2}{\left(\theta_{ijk}-\theta_{0}\right)}^{2}$$

for each triad of consecutive particles when *k*_*A*_ = 5 and θ_0_ = 180°. To compute long-range electrostatic interactions, the same parts per million method was used. This method has been shown to be a suitable choice for MDPD simulations and does not present artificial ion pair formation.^39^

#### Mapping to Real Units

To map MDPD reduced units to real units, we follow the original MDPD–Martini FF parametrization, which used the same scaling approach as in prior MDPD water parametrization studies at a coarse-graining level of three water molecules per bead.^40,87^

All unit conversions used are shown in Table 3. In MDPD, the typical length scale is set by attractive interaction cutoff *r*_c_ and by the coarse-graining level. Its real value is obtained by comparing the density of beads in a simulation with the real molecular volume of water (*V* = 30 Å^3^). In a similar manner, the density can be converted by using the molecular mass of water (*M* = 18 g/mol) and Avogadro’s number (*N*_A_). The energy scale in MDPD is set by the value of *k*_B_ *T*, where *k*_B_ is the Boltzmann constant and *T* the temperature.

**Table 3:** Conversion between the MDPD and Real Units^a^

parameter	MDPD	conversion	MD
*r*_c_	1	(*ρN*_m_ *V*)^1/3^	8.53 Å
ρ	6.9	*ρN*_m_ *M*/*N*_A_ *r*_c_^3^	997 kg/m^3^
γ	12.4	*γk*_B_ *T*/*r*_c_^2^	72 mN/m
*Δt*	0.01	*N*_m_ *D*_MDPD_ *r*_c_^2^/*D*_water_	0.43 ps

a The values presented are for the parametrization of water with a coarse-graining level (*N*_m_) of 3. The MDPD values can be obtained from a bulk water simulation with the following values: *A* = −50, *B* = 25, *r*_c_ = 1, and *r*_d_ = 0.75. Length scale *r*_c_ is converted by using the volume of a water molecule, that is, *V* = 30 Å^3^. Time is scaled by the diffusion coefficient of water (*D*_water_). The density conversion uses the molar mass of water (*M*) and Avogadro’s number (*N*_A_).

Time scaling is obtained by conducting pure bulk water simulations and measuring the MDPD self-diffusivity (*D*_MDPD_) from the mean square displacement of the beads (*D*_MDPD_), which for the water model used is 0.049.^88^ Using a time step (*Δt*) of 0.01, the conversion shows that this corresponds to 430 fs, which is 20 times longer than the time step of 20 fs required in our MD simulations. A comparison of the computational wall time between MD and MDPD can be found in the Supporting Information.

### Systems

We carried out two different sets of simulations to compare different SDS properties. In the first case, aiming to determine interfacial properties, we have defined a simulation box with dimensions *L*_*x*_ = *L*_*y*_ = 17 nm and *L*_*z*_ = 60 nm. In the middle of the simulation box, we placed a water slab with a thickness of 17 nm with interface normals in the *z* direction. Surfactant molecules were then placed on the interfaces, while an equal amount of counterions was placed in the water slab. A representation of such a system is shown in Figure 2a. Different initial surfactant surface coverages (*C*) were simulated with the number of molecules ranging from 160 to 1440 in the MDPD case and from 160 to 960 in the MD case. The surface coverage is defined as the total number of surfactants divided by the total interfacial area in the simulation, and for systems below the CAC, the surface coverage will be approximately equal to the surface excess concentration, as most surfactants will stay at the interface. This set of simulations was done on the canonical ensemble (*NVT*), and systems were allowed to equilibrate for 10^6^ time steps while their energy was monitored to ensure they have reached an equilibrium (see the Supporting Information), followed by a production run with 10^6^ time steps, where the surface tension was measured and averaged over time. For MDPD simulations, the equations of motion in the *NVT* ensemble are integrated by a modified velocity–Verlet algorithm, which is the standard approach.^69,70,89^

**Figure 2:**
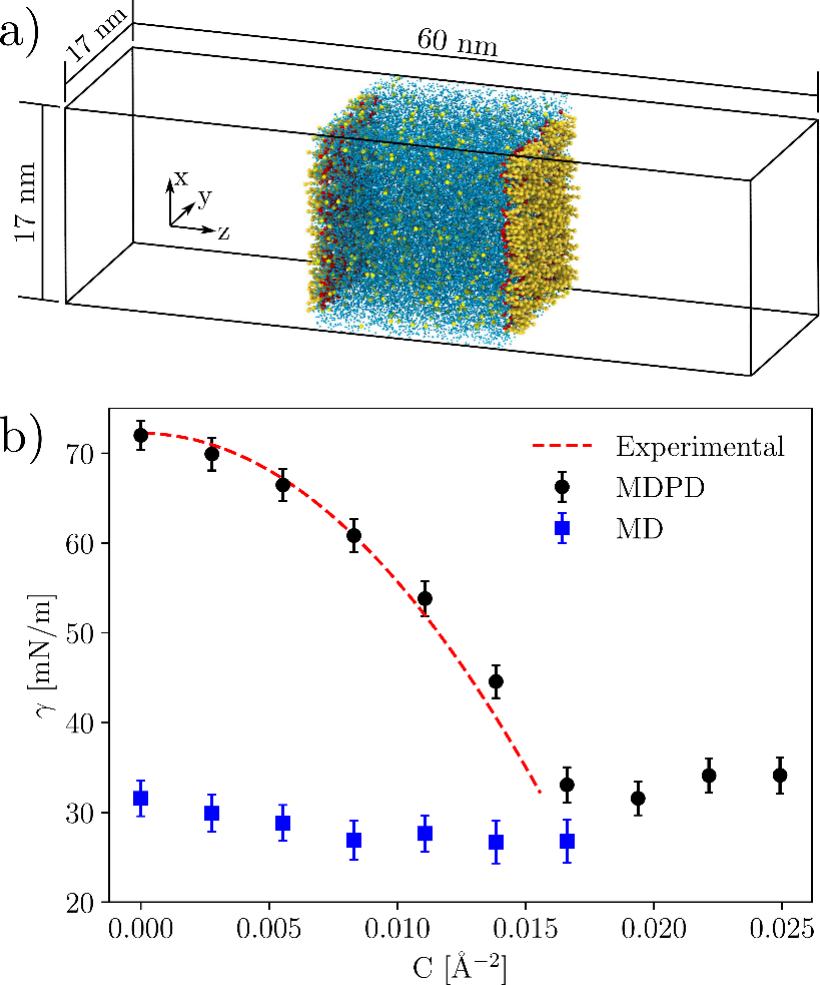
(a) Typical system setup for calculating the surface tension in the simulations. The same system parameters were used for both MD and MDPD simulations. Water beads are represented with a smaller radius for the purpose of visualization. The color code follows the model presented in Figure 1. A snapshot has been obtained using OVITO.^90^ (b) Surface tension isotherms from MD and MDPD simulations and an experiment^67^ (see the text for details), as indicated. The horizontal axis reflects the values of the surface excess concentration, while γ is the surface tension.

In the second set of simulations, we focused on SDS bulk properties. All systems were composed of 1000 surfactant molecules, 1000 sodium ions, and enough water beads to reproduce the desired concentration. Because the water parametrization used in the development of the MDPD FF follows a 3:1 mapping, the number of water beads in the MDPD simulations was higher than that in the MD simulations, at a ratio of 4:3. In a system with 45% SDS, for example, we used 3889 and 5185 water beads in the MD and MDPD simulations, respectively. The number of water beads for different concentrations can be found in the Supporting Information. The molecules were placed in a random distribution inside a cubic simulation box, and an initial energy minimization step was performed to avoid any numerical instabilities caused by possible overlap of beads in the initial configuration. The minimization was done by a conjugate gradient method with the tolerance set to 10^–6^. This set of simulations was done on the isothermal–isobaric (*NPT*) ensemble and ran for 3 × 10^6^ time steps, which was enough to reach equilibrium based on measurements of the total energy of the system. To represent the *NPT* ensemble in MDPD, we chose to integrate the equations of motion using the isobaric–isoenthalpic (*NPH*) integrator in LAMMPS, which controls the pressure in the simulation, while the temperature was still controlled by the MDPD thermostat, i.e., via the **F** ^R^ and **F** ^D^ forces.

## Results and Discussion

### Interfacial Properties

The surface tension isotherm for the water–SDS–air interface can be calculated in the simulations through the mechanical route by using the setup of Figure 2a.^91^ This is a relevant property as surfactants adsorb on the interface, reducing the interfacial tension, and could change the dynamics of different phenomena, such as droplet coalescence and breakup.^8,42^ Our simulations involve a slab of water laden with SDS molecules with periodic boundary conditions applied in all directions of the simulation box. While the dimensions of the liquid slab coincide with the size of the liquid phase in the directions parallel to the free surface (*x* and *y*), the box size in the direction normal to the free surface (*z* (Figure 2a)) is larger. The number of water beads added to the slab region was obtained from the average density of beads in bulk water simulations for both MD and MDPD. Through this system setup, the pressure difference parallel and perpendicular to the flat, free surface (liquid–vapor surface) would reflect the magnitude of the surface tension due to the presence of the liquid–vapor surface. This is expressed mathematically as follows:

11 $$\gamma =\frac{L_{z}}{2}\left(P_{zz}-\frac{P_{xx}+P_{yy}}{2}\right)$$

which in fact accounts for the presence of two liquid–vapor surfaces in the system and the integration being throughout the simulation box (*L*_*z*_) in the *z* direction. To compare these results with surface tension isotherms obtained through experiments,^67^ one needs to replace the bulk surfactant concentration with the surface excess concentration, which is done by using the following relation^92^

12 $$C=-\frac{1}{RT}\frac{d\gamma }{d\;\ln c}$$

where *R* is the gas constant, *c* the bulk surfactant concentration, γ the surface tension, and *C* the surface excess concentration. It should be noted that this relation should in principle hold for a charge neutral surfactant at a given constant temperature, *T*, and pressure, *P*, and is valid in the pre-CAC region, for which the $\frac{d\gamma }{d\;\ln c}$ slopes can be calculated. Thus, one can produce experimental curves in the form obtained by eq 12, making a comparison between simulation and experimental results possible.^9,92^ For a charged system, eq 12 has a correction factor that depends on the ionic activity coefficients. However, because the bulk concentration of surfactants in the pre-CAC region is low and we have a 1:1 counterion:surfactant ratio, we can apply the usual Gibbs adsorption isotherm.^93^ The isotherm obtained through the MDPD–Martini model (Figure 2) shows a very good agreement with the experimental data, which are indicated by the red curve.^67^ In contrast, the MD–Martini model deviates considerably from the experimental and MDPD–Martini surface tension isotherms. Moreover, the MD–Martini model exhibits significant deviations from the benchmark value of about 72 mN/m for water.^67,94^ In addition, the surface tension at a concentration near the CAC is underestimated in the MD–Martini model (Figure 2), in comparison with a value of about 35 mN/m found in the case of the MDPD–Martini model and experiment.^67^ Hence, the MDPD–Martini model seems to offer advantages when investigating phenomena where surface tension is expected to play an important role, such as droplet coalescence,^95^ breakup,^96^ and oscillation phenomena.^97^

We further explored the interfacial properties of the SDS/water systems by calculating the density distribution of the various chemical groups at the surface, juxtaposing the MD and MDPD results. In this case, computer simulation is particularly suitable for elucidating the surface structure at molecular resolution. While the MD–Martini and MDPD–Martini models are in good agreement, slight differences can be observed. Considering a typical case for a specific surface coverage (*C*) of 0.0055 Å^–2^ (Figure 3), we find slightly broader distributions in the case of MD than in the MDPD results, which may indicate that the surfactant molecules in the MDPD model are more localized at the liquid–vapor surface. This is also suggested by the fact that the water density distribution shows slightly larger deviations from the total density for all components in the case of MD simulations. These observations may indicate an overall more well-defined structure of the interface in the case of MDPD–Martini models than in the MD–Martini model, despite the soft nature of interactions in the MDPD–Martini FF.

**Figure 3:**
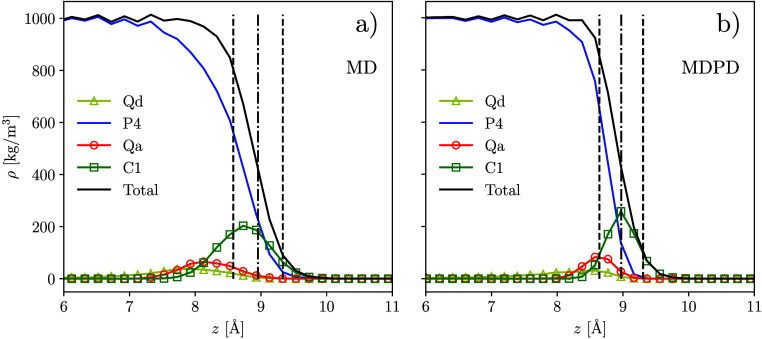
Density distribution of different beads across the liquid–vapor surface for (a) MD and (b) MDPD simulations. These distributions were obtained for an initial surface coverage (*C*) of 0.0055 Å^–2^, which is below the concentration at which micelles start to form for both methods.

The sigmoidal shape of the total density as a function of distance *z* (Figure 3) is often fitted to the following relation

13 $$\rho \left(z\right)=\frac{\rho_{l}}{2}\left[1-\tanh\left(\frac{2\left(z-z_{0}\right)}{\delta }\right)\right]$$

whence the thickness of the liquid–vapor surface (δ) can be obtained. Typically, it is expected that surfactant molecules will pack differently as their concentration at the liquid–vapor surface increases, which is manifested by a change in the slope when the thickness (δ) is plotted versus the surface excess concentration (*C*).^43,96^ This effect clearly appears in the plot in Figure 4. In the case of the MDPD–Martini model, initially the rate of thickness increase is 405 Å^–1^, which later decreases for higher values of the surface excess concentration as the liquid–vapor surface saturates. The maximum thickness (δ) recorded is about 11 Å. In contrast, the MD–Martini data indicate a smaller slope for the thickness, which saturates to a maximum value, namely, 7.5 Å, at a lower surface excess concentration in comparison with the MDPD data. Hence, the MD data obtained for the δ seem to be less sensitive to the variation in the surface excess concentration, in contrast to the data based on the MDPD–Martini model.

**Figure 4:**
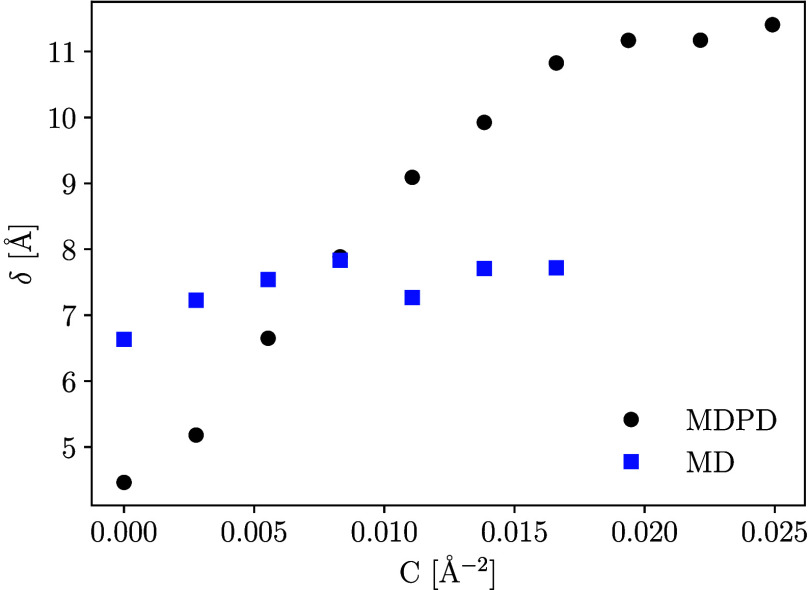
Thicknesses (δ) of the liquid–vapor surface as a function of surface excess concentration for MD and MDPD methods, as indicated. δ is obtained through eq 13.

### Bulk Behavior

We now turn our attention to the various bulk properties of the system. One property of interest here is the aggregation number (*N*_agg_),^83^ which can be calculated as follows

14 $$N_{\mathrm{agg}}=\frac{\underset{i}{\sum }{N_{i}}^{2}P\left(N_{i}\right)}{\underset{i}{\sum }N_{i}P\left(N_{i}\right)}$$

where *P*(*N*) is the aggregation number distribution while *N* is the number of molecules in a given micelle. This number is calculated when the distribution has reached a constant value, and the peak average value does not shift. Moreover, the final value is calculated from three independent trajectories following the procedure of previous MD–Martini simulations.^33^ The monomers belonging to each micelle have been determined by applying a cluster algorithm with the distance chosen based on the Stillinger criterion.^98^ A histogram presenting the aggregation number distribution for both MD and MDPD simulations is available in the Supporting Information. In the case of MD–Martini simulations, *N*_agg_ = 50 ± 13, which is lower than expected for the given concentration (*c* = 25%).^68^ The corresponding value in the case of the MDPD–Martini model is 55 ± 21. MDPD results showed a larger deviation from the average, but the two values are in good agreement with each other The underprediction of aggregation numbers from the standard MD–Martini version 2.2, which was used in this paper, is a known issue.^28,71,99^ To properly reproduce the self-assembly of SDS micelles, modifications to the standard force field or the use of a slightly different force field, such as having implicit solvent models, is usually necessary.^33^ It is worth noting that the more recent MD–Martini version 3.0 can achieve results comparable to experimental values,^45,100^ and we expect future versions of MDPD–Martini to be able to improve in the same direction as more interaction levels and beads are parametrized.

The next property that we have examined is the morphology of various SDS–water systems for different surfactant concentrations above the CAC. The expected morphologies for the SDS/water system are micelles, hexagonal and lamellar.^101^ These have been confirmed by MD–Martini simulations,^33^ and we have also reproduced here the MD–Martini results for the same concentrations, which are presented in Figure 5. The MDPD–Martini model can reproduce these morphologies in line with the MD–Martini^33^ results and experiments.^101^ In Figure 5, the number of micelles is 18 for MDPD versus 20 for MD. The average distance between the cylinders in the hexagonal phase is about 43.4 Å in MDPD versus 39.8 Å in MD, while the lamellar width is 31.8 Å in MDPD versus 28.7 Å in MD.

**Figure 5:**
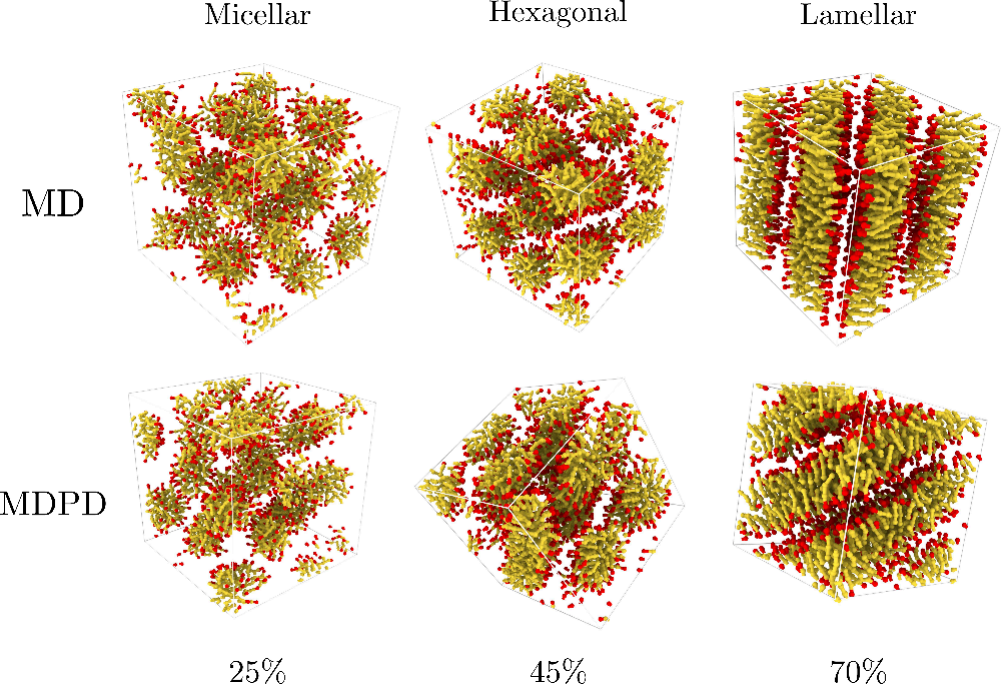
Different surfactant phases as a function of concentration. Both MD and MDPD can reproduce the same behavior, in line with experiments.^101^ These simulations were done with 1000 SDS molecules and 1000 counterions, while the number of water beads was adjusted for the desired concentration. Only surfactants are shown for better visualization of the morphologies. Snapshots were obtained by using OVITO.^90^

### Coherent Scattered Intensity

We have examined the scattering pattern of an SDS solution at 5% and 25%, which can be compared with SANS experimental results.^68^ In the simulations, this can be obtained through the coherent scattered intensity from the Fourier transform of radial distribution functions *g*_*ij*_(*r*) expressed as follows

15 $$I\left(q\right)=\overset{n}{\underset{i=1}{\sum }}\overset{n}{\underset{j=1}{\sum }}x_{i}x_{j}f_{i}f_{j}\left(1+\rho \int_{0}^{\infty }4\pi r^{2}\left(g_{ij}\left(r\right)-1\right)\frac{\sin\left(qr\right)}{qr}\;dr\right)$$

where *n* is the number of different bead types, *x*_*i*_ is the concentration of beads of type *i*, *f*_*i*,*j*_ are the atomic form factors, and *g*_*ij*_(*r*) is the radial distribution function between beads of types *i* and *j*.

To allow for a direct comparison with previous data,^33^ which are also here reproduced for different surfactant concentrations in the micelle regime and take into account the coarse-grained nature of the MD and MDPD models, we set *f*_*i*_ = *f*_*j*_ = 1. The coherent scattered intensity for solutions with different surfactant concentrations as indicated is presented in Figure 6. The MD and MDPD data are in very good agreement for both concentrations shown here. The position of the peak approximately reflects the mean value of the distance between the micelles, which decreases as the surfactant concentration increases.^39^ These distances correspond to a λ of ≈5.7 nm for a surfactant concentration of 5%, while λ ≈ 4.5 nm for a concentration of 25%, where λ = 2π/*q*. The experimental values are 6.9 nm for a concentration 20% and 8.6 nm for a concentration of 5% at 293 K. Hence, both MD and MDPD data generally underpredict the wavelength of the peak intensity. This difference is due to the lower aggregation numbers obtained in our simulations. A relation between the aggregation number and the mean micelle distance can be obtained from the intermicellar Coulombic repulsion model (ICRM)^102^ and is given by the equation *N*_agg_ = *cN*_A_λ^3^, where *c* is the molar concentration and *N*_A_ is Avogadro’s number. Our results match with the estimates from this equation for both concentrations. In addition, the shapes of the curves for both MD and MDPD simulations are the same. In comparison with experimental data,^68^ certain differences are expected at high wavelengths, due to the coarse-grained character of the simulation models, which has been discussed previously.^33^

**Figure 6:**
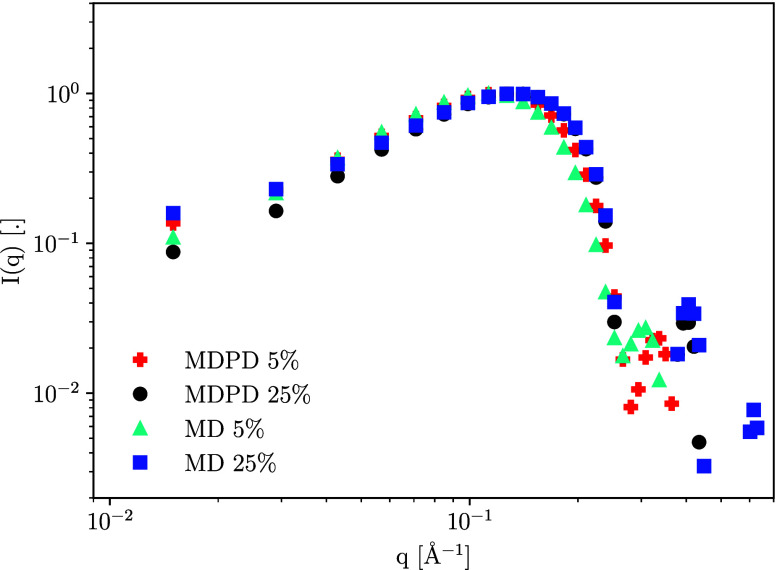
Coherent scattered intensity vs wavenumber (*q*). The wavelength can be obtained through the expression λ = 2π/*q*. Both MD and MDPD present the same peaks at their respective concentrations. For 5%, λ ≈ 5.7 nm, while for 25%, λ ≈ 4.5 nm.

## Conclusions

In this study, we have presented the MDPD–Martini model for the SDS surfactant, which is capable of describing several properties of water/SDS systems. Moreover, a direct comparison between the MDPD–Martini and MD–Martini models has been conducted with results for MD simulations from the literature^33^ reproduced in our study. Our analysis indicated that the MD–Martini and MDPD–Martini models are in very good agreement with each other and with experimental results. Moreover, the MDPD–Martini model can better reproduce the surface tension isotherm, in line with the experimental data.^67^ The results are also in line with a previous MDPD model for the SDS surfactant.^39^

The MDPD–Martini model presented here relies on transferable interactions^46,47^ and explicitly modeling the cations as point charges as separate beads, an approach that has been only recently employed in MDPD models.^39^ A current limitation of our force field is the small number of parametrized beads and interaction levels, which reduces the scope of chemical specificity and the number of possible molecule representations. However, further steps in the development of the general-purpose MDPD–Martini model will include additions to the current interaction matrix based on the Martini mapping,^45^ as well as the integration of methodologies, such as those related to reactivity^65^ and the Go̅Martini approach.^66^ In view of the computational acceleration provided by the MDPD–Martini models in comparison with MD,^46^ we anticipate that the current study opens opportunities for further applications of the MDPD–Martini models to a greater range of soft-matter systems, including systems that require the explicit modeling of charges. Finally, our study can provide further inspiration for the development of a general-purpose force field based on non-LJ interactions.

## Supplementary Material



## Notes

Additional figures on energy equilibration, micelle aggregation, and surface tension measurements and tables of system descriptions and computational time comparisons (PDF)

## References

[1] Broze, G. , Ed. Handbook of Detergents; CRC Press: Boca Raton, FL, 1999.

[2] Becher, P. , Ed. Emulsion: Theory and Practice; Reinhold Press: New York, 1965.

[3] Goodarzi F., Zendehboudi S. (2019). A Comprehensive Review on Emulsions
and Emulsion Stability
in Chemical and Energy Industries. Can. J. Chem.
Eng..

[4] Rosen, M. J. , Kunjappu, J. T. , Eds. Surfactants and interfacial phenomena; John Wiley & Sons, Inc.: Hoboken, NJ, 2012.

[5] Blute I., Jansson M., Oh S. G., Shah D. O. (1994). The molecular mechanism
for destabilization of foams by organic ions. J. Am. Oil Chem. Soc..

[6] Lunkenheimer K., Lind A., Jost M. (2003). Surface Tension of Surfactant Solutions. J. Phys. Chem. B.

[7] Berthod A., Tomer S., Dorsey J. G. (2001). Polyoxyethylene alkyl ether nonionic
surfactants: physicochemical properties and use for cholesterol determination
in food. Talanta.

[8] Arbabi S., Deuar P., Denys M., Bennacer R., Che Z., Theodorakis P. E. (2023). Molecular dynamics simulation of
the coalescence of
surfactant-laden droplets. Soft Matter.

[9] Theodorakis P. E., Müller E. A., Craster R. V., Matar O. K. (2015). Superspreading:
Mechanisms and Molecular Design. Langmuir.

[10] Missel P. J., Mazer N. A., Benedek G. B., Young C. Y., Carey M. C. (1980). Thermodynamic
analysis of the growth of sodium dodecyl sulfate micelles. J. Phys. Chem..

[11] Cooper R. S. (1963). Anionic
phosphate surfactants. J. Am. Oil Chem. Soc..

[12] Boethling, R. S. ; Lynch, D. G. In Detergents; de Oude, N. T. , Ed.; Springer: Berlin, 1992; pp 145–177.

[13] FernLey G. W. (1978). Zwitterionic
surfactants: Structure and performance. J. Am.
Oil Chem. Soc..

[14] Cabane B., Duplessix R., Zemb T. (1985). High resolution neutron scattering
on ionic surfactant micelles: sds in water. J. Phys. (Paris).

[15] Bergström M., Skov Pedersen J. (1999). Structure
of pure SDS and DTAB micelles in brine determined
by small-angle neutron scattering (SANS). Phys.
Chem. Chem. Phys..

[16] Magid L. J., Li Z., Butler P. D. (2000). Flexibility of Elongated Sodium Dodecyl Sulfate Micelles
in Aqueous Sodium Chloride: A Small-Angle Neutron Scattering Study. Langmuir.

[17] Kakitani M., Imae T., Furusaka M. (1995). Investigation of Mixed Micelles of
Dodecyldimethylamine Oxide and Sodium Dodecyl Sulfate by SANS: Shape,
Size, Charge, and Interaction. J. Phys. Chem..

[18] Ludwig M., Geisler R., Prévost S., von Klitzing R. (2021). Shape and
Structure Formation of Mixed Nonionic-Anionic Surfactant Micelles. Molecules.

[19] Khodaparast S., Sharratt W. N., Tyagi G., Dalgliesh R. M., Robles E. S., Cabral J. T. (2021). Pure and mixed aqueous
micellar solutions
of Sodium Dodecyl sulfate (SDS) and Dimethyldodecyl Amine Oxide (DDAO):
Role of temperature and composition. J. Colloid
Interface Sci..

[20] Bezzobotnov V. Y., Borbely S., Cser L., Farago B., Gladkih I. A., Ostanevich Y. M., Vass S. (1988). Temperature and Concentration
Dependence
of Properties of Sodium Dodecyl Sulfate Micelles Determined from Small
Angle Neutron Scattering Experiments. J. Phys.
Chem..

[21] Gawali S. L., Zhang M., Kumar S., Ray D., Basu M., Aswal V. K., Danino D., Hassan P. A. (2019). Discerning the Structure
Factor of Charged Micelles in Water and Supercooled Solvent by Contrast
Variation X-ray Scattering. Langmuir.

[22] Lipfert J., Columbus L., Chu V. B., Lesley S. A., Doniach S. (2007). Size and Shape
of Detergent Micelles Determined by Small-Angle X-ray Scattering. J. Phys. Chem. B.

[23] Aniansson E. A. G., Wall S. N., Almgren M., Hoffmann H., Kielmann I., Ulbricht W., Zana R., Lang J., Tondre C. (1976). Theory of
the kinetics of micellar equilibria and quantitative interpretation
of chemical relaxation studies of micellar solutions of ionic surfactants. J. Phys. Chem..

[24] Quina F. H., Nassar P. M., Bonilha J. B. S., Bales B. L. (1995). Growth of Sodium
Dodecyl Sulfate Micelles with Detergent Concentration. J. Phys. Chem..

[25] Dutkiewicz E., Jakubowska A. (2002). Effect of electrolytes on the physicochemical behaviour
of sodium dodecyl sulphate micelles. Colloid
Polym. Sci..

[26] Khan H., Seddon J. M., Law R. V., Brooks N. J., Robles E., Cabral J. T., Ces O. (2019). Effect of glycerol with sodium chloride
on the Krafft point of sodium dodecyl sulfate using surface tension. J. Colloid Interface Sci..

[27] Yatsyshin P., Fytas N. G., Theodorakis P. E. (2020). Mixing–demixing
transition
in polymer-grafted spherical nanoparticles. Soft Matter.

[28] Peroukidis S. D., Mintis D. G., Stott I., Mavrantzas V. G. (2021). Molecular
simulation of the morphology and viscosity of aqueous micellar solutions
of sodium lauryl ether sulfate (SLEnS). J. Phys.
Mater..

[29] Tang X., Koenig P. H., Larson R. G. (2014). Molecular
Dynamics Simulations of
Sodium Dodecyl Sulfate Micelles in Water—The Effect of the
Force Field. J. Phys. Chem. B.

[30] Jalili S., Akhavan M. (2009). A coarse-grained molecular
dynamics simulation of a
sodium dodecyl sulfate micelle in aqueous solution. Colloids Surf. A: Physicochem. Eng. Asp..

[31] Palazzesi F., Calvaresi M., Zerbetto F. (2011). A molecular dynamics investigation
of structure and dynamics of SDS and SDBS micelles. Soft Matter.

[32] Chun B. J., Choi J. I., Jang S. S. (2015). Molecular dynamics simulation study
of sodium dodecyl sulfate micelle: Water penetration and sodium dodecyl
sulfate dissociation. Colloids Surf. A: Physicochem.
Eng. Asp..

[33] Anogiannakis S. D., Petris P. C., Theodorou D. N. (2020). Promising Route for the Development
of a Computational Framework for Self-Assembly and Phase Behavior
Prediction of Ionic Surfactants Using MARTINI. J. Phys. Chem. B.

[34] Zhou J., Ranjith P. (2021). Self-assembly and viscosity changes
of binary surfactant
solutions: A molecular dynamics study. J. Colloid
Interface Sci..

[35] Wang H., Zhang H., Liu C., Yuan S. (2012). Coarse-grained
molecular
dynamics simulation of self-assembly of polyacrylamide and sodium
dodecylsulfate in aqueous solution. J. Colloid
Interface Sci..

[36] Anderson R. L., Bray D. J., Del Regno A., Seaton M. A., Ferrante A. S., Warren P. B. (2018). Micelle Formation
in Alkyl Sulfate Surfactants Using
Dissipative Particle Dynamics. J. Chem. Theory
Comput..

[37] Mao R., Lee M.-T., Vishnyakov A., Neimark A. V. (2015). Modeling Aggregation
of Ionic Surfactants Using a Smeared Charge Approximation in Dissipative
Particle Dynamics Simulations. J. Phys. Chem.
B.

[38] Gray S. J., Walker M., Hendrikse R., Wilson M. R. (2023). Investigating anionic
surfactant phase diagrams using dissipative particle dynamics: development
of a transferable model. Soft Matter.

[39] Hendrikse R. L., Amador C., Wilson M. R. (2024). Many-body
dissipative particle dynamics
simulations of micellization of sodium alkyl sulfates. Soft Matter.

[40] Ghoufi A., Emile J., Malfreyt P. (2013). Recent Advances in Many Body Dissipative
Particles Dynamics Simulations of Liquid-Vapor Interfaces. Eur. Phys. J. E.

[41] Zhou P., Hou J., Yan Y., Wang J., Chen W. (2019). Effect of Aggregation
and Adsorption Behavior on the Flow Resistance of Surfactant Fluid
on Smooth and Rough Surfaces: A Many-Body Dissipative Particle Dynamics
Study. Langmuir.

[42] Carnevale L. H., Deuar P., Che Z., Theodorakis P. E. (2024). Surfactant-laden
liquid thread breakup driven by thermal fluctuations. Phys. Fluids.

[43] Hendrikse R. L., Amador C., Wilson M. R. (2023). A many-body
dissipative particle
dynamics parametrisation scheme to study behaviour at air-water interfaces. Soft Matter.

[44] Marrink S. J., de Vries A. H., Mark A. E. (2004). Coarse Grained Model for Semiquantitative
Lipid Simulations. J. Phys. Chem. B.

[45] Souza P. C. T. (2021). Martini 3: a general
purpose force field for coarse-grained
molecular dynamics. Nat. Methods.

[46] Carnevale L. H., Theodorakis P. E. (2024). Many-body
disiipative particle dynamics with the MARTINI
LEGO approach. Eur. Phys. J. Plus.

[47] Kramarz N., Carnevale L. H., Theodorakis P. E. (2025). Many-body dissipative particle dynamics
simulations of lipid bilayers with the MDPD-MARTINI force-field. Eur. Phys. J. Plus.

[48] Marrink S. J., Risselada H. J., Yefimov S., Tieleman D. P., de Vries A. H. (2007). The MARTINI
Force Field: Coarse Grained Model for Biomolecular Simulations. J. Phys. Chem. B.

[49] Marrink S. J., Tieleman D. P. (2013). Perspective on the Martini model. Chem. Soc. Rev..

[50] Alessandri R., Grünewald F., Marrink S. J. (2021). The Martini Model in Materials Science. Adv. Mater..

[51] Zhao J., Chen S., Zhang K., Liu Y. (2021). A review of many-body
dissipative particle dynamics (MDPD): Theoretical models and its applications. Phys. Fluids.

[52] Souza R. C. T. (2025). Go̅Martini 3:
From large conformational changes
in proteins to environmental bias corrections. Nat. Commun..

[53] Monticelli L., Kandasamy S. K., Periole X., Larson R. G., Tieleman D. P., Marrink S.-J. (2008). The MARTINI
Coarse-Grained Force Field: Extension to
Proteins. J. Chem. Theory Comput..

[54] Periole X., Cavalli M., Marrink S.-J., Ceruso M. A. (2009). Combining an Elastic
Network With a Coarse-Grained Molecular Force Field: Structure, Dynamics,
and Intermolecular Recognition. J. Chem. Theory
Comput..

[55] de
Jong D. H., Singh G., Bennett W. F. D., Arnarez C., Wassenaar T. A., Schäfer L. V., Periole X., Tieleman D. P., Marrink S. J. (2013). Improved Parameters for the Martini Coarse-Grained
Protein Force Field. J. Chem. Theory Comput..

[56] Lee H., de Vries A. H., Marrink S.-J., Pastor R. W. (2009). A Coarse-Grained
Model for Polyethylene Oxide and Polyethylene Glycol: Conformation
and Hydrodynamics. J. Phys. Chem. B.

[57] López C. A., Rzepiela A. J., de Vries A. H., Dijkhuizen L., Hünenberger P.
H., Marrink S. J. (2009). Martini
Coarse-Grained
Force Field: Extension to Carbohydrates. J.
Chem. Theory Comput..

[58] López C. A., Sovova Z., van Eerden F. J., de Vries A. H., Marrink S. J. (2013). Martini
Force Field Parameters for Glycolipids. J. Chem.
Theory Comput..

[59] Chakraborty S., Wagh K., Gnanakaran S., López C. A. (2021). Development
of Martini 2.2 parameters for N-glycans: a case study of the HIV-1
Env glycoprotein dynamics. Glycobiology.

[60] Uusitalo J. J., Ingólfsson H. I., Akhshi P., Tieleman D. P., Marrink S. J. (2015). Martini
Coarse-Grained Force Field: Extension to DNA. J. Chem. Theory Comput..

[61] Uusitalo J. J., Ingólfsson H.
I., Marrink S. J., Faustino I. (2017). Martini Coarse-Grained
Force Field: Extension to RNA. Biophys. J..

[62] Yesylevskyy S. O., Schäfer L. V., Sengupta D., Marrink S. J. (2010). Polarizable Water
Model for the Coarse-Grained MARTINI Force Field. PLoS Comput. Biol..

[63] Vainikka P., Thallmair S., Souza P. C. T., Marrink S. J. (2021). Martini 3 Coarse-Grained
Model for Type III Deep Eutectic Solvents: Thermodynamic, Structural,
and Extraction Properties. ACS Sustain. Chem.
Eng..

[64] Grünewald F., Souza P. C. T., Abdizadeh H., Barnoud J., de Vries A. H., Marrink S. J. (2020). Titratable Martini model for constant pH simulations. J. Chem. Phys..

[65] Sami S., Marrink S. J. (2023). Reactive Martini:
Chemical Reactions in Coarse-Grained
Molecular Dynamics Simulations. J. Chem. Theory
Comput..

[66] Poma A. B., Cieplak M., Theodorakis P. E. (2017). Combining the MARTINI and Structure-Based
Coarse-Grained Approaches for the Molecular Dynamics Studies of Conformational
Transitions in Proteins. J. Chem. Theory Comput..

[67] Fainerman V., Lylyk S., Aksenenko E., Petkov J., Yorke J., Miller R. (2010). Surface tension isotherms,
adsorption dynamics and
dilational visco-elasticity of sodium dodecyl sulphate solutions. Colloids Surf. A Physicochem. Eng. Asp..

[68] Hammouda B. (2013). Temperature
Effect on the Nanostructure of SDS Micelles in Water. J. Res. Natl. Inst. Stand. Technol..

[69] Plimpton S. (1995). Fast Parallel
Algorithms for Short-Range Molecular Dynamics. J. Comput. Phys..

[70] Thompson A. P., Aktulga H. M., Berger R., Bolintineanu D. S., Brown W. M., Crozier P. S., in ’t Veld P. J., Kohlmeyer A., Moore S. G., Nguyen T. D., Shan R., Stevens M. J., Tranchida J., Trott C., Plimpton S. J. (2022). LAMMPS
- a flexible simulation tool for particle-based materials modeling
at the atomic, meso, and continuum scales. Comput.
Phys. Commun..

[71] Wang S., Larson R. G. (2015). Coarse-Grained Molecular Dynamics Simulation of Self-Assembly
and Surface Adsorption of Ionic Surfactants Using an Implicit Water
Model. Langmuir.

[72] Español P., Warren P. (1995). Statistical Mechanics
of Dissipative Particle Dynamics. Europhys.
Lett. (EPL).

[73] Warren P. B. (2003). Vapor-liquid
coexistence in many-body dissipative particle dynamics. Phys. Rev. E.

[74] Zhao J., Chen S., Phan-Thien N. (2018). Viscometric flow for a many-body
dissipative particle dynamics (MDPD) fluid with Lees–Edwards
boundary condition. Mol. Sim..

[75] Zhao C., Zhao J., Si T., Chen S. (2021). Influence of thermal
fluctuations on nanoscale free-surface flows: A many-body dissipative
particle dynamics study. Phys. Fluids.

[76] Han Y., Jin J., Voth G. A. (2021). Constructing
many-body dissipative particle dynamics
models of fluids from bottom-up coarse-graining. J. Chem. Phys..

[77] Vanya P., Crout P., Sharman J., Elliott J. A. (2018). Liquid-phase parametrization
and solidification in many-body dissipative particle dynamics. Phys. Rev. E.

[78] Carnevale L. H., Deuar P., Che Z., Theodorakis P. E. (2023). Liquid
thread breakup and the formation of satellite droplets. Phys. Fluids.

[79] Theodorakis P. E., Paul W., Binder K. (2011). Analysis of
the cluster formation
in two-component cylindrical bottle-brush polymers under poor solvent
conditions. A simulation study. Eur. Phys. J.
E.

[80] Español P., Warren P. B. (2017). Perspective: Dissipative
particle dynamics. J. Chem. Phys..

[81] Yoshimoto Y., Kinefuchi I., Mima T., Fukushima A., Tokumasu T., Takagi S. (2013). Bottom-up
construction of interaction
models of non-Markovian dissipative particle dynamics. Phys. Rev. E.

[82] Li Z., Bian X., Caswell B., Karniadakis G. E. (2014). Construction
of dissipative particle dynamics models for complex fluids via the
Mori–Zwanzig formulation. Soft Matter.

[83] Lavagnini E., Cook J. L., Warren P. B., Hunter C. A. (2021). Translation of Chemical
Structure into Dissipative Particle Dynamics Parameters for Simulation
of Surfactant Self-Assembly. J. Phys. Chem.
B.

[84] Trofimov S. Y., Nies E. L. F., Michels M. A. J. (2005). Constant-pressure simulations with
dissipative particle dynamics. J. Chem. Phys..

[85] Groot R. D., Warren P. B. (1997). Dissipative particle
dynamics: Bridging the gap between
atomistic and mesoscopic simulation. J. Chem.
Phys..

[86] Warren P.
B. (2013). No-go theorem
in many-body dissipative particle dynamics. Phys. Rev. E.

[87] Ghoufi A., Malfreyt P. (2011). Mesoscale modeling
of the water liquid-vapor interface:
A surface tension calculation. Phys. Rev. E.

[88] Deng X., Müller-Plathe F. (2025). Underwater
Adhesion of Oil Droplets to Surfaces Grafted
with Hydrophilic Polymer Brushes: Many-Body Dissipative-Particle Simulations. Langmuir.

[89] Pagonabarraga I., Frenkel D. (2001). Dissipative particle dynamics for
interacting systems. J. Chem. Phys..

[90] Stukowski A. (2010). Visualization and
analysis of atomistic simulation data with OVITO-the Open Visualization
Tool.

[91] Kirkwood J. G., Buff F. P. (1949). The Statistical
Mechanical Theory of Surface Tension. J. Chem.
Phys..

[92] Herdes C., Santiso E. E., James C., Eastoe J., Müller E. A. (2015). Modelling
the interfacial behaviour of dilute light-switching surfactant solutions. J. Colloid Interface Sci..

[93] Martínez-Balbuena L., Arteaga-Jiménez A., Hernández-Zapata E., Márquez-Beltrán C. (2017). Applicability
of the Gibbs Adsorption
Isotherm to the analysis of experimental surface-tension data for
ionic and nonionic surfactants. Adv. Colloid
Interface Sci..

[94] Iannetti L., Cambiaso S., Rasera F., Giacomello A., Rossi G., Bochicchio D., Tinti A. (2024). The surface tension
of Martini 3 water mixtures. J. Chem. Phys..

[95] Arbabi S., Deuar P., Denys M., Bennacer R., Che Z., Theodorakis P. E. (2023). Coalescence
of surfactant-laden droplets. Phys. Fluids.

[96] Carnevale L. H., Deuar P., Che Z., Theodorakis P. E. (2024). Surfactant-laden
liquid thread breakup driven by thermal fluctuations. Phys. Fluids.

[97] Ng K. L., Carnevale L. H., Klamka M., Deuar P., Bobinski T., Theodorakis P. E. (2025). Oscillations
of a water droplet on a horizontally vibrating
substrate. Phys. Fluids.

[98] Stillinger F. H. (1963). Rigorous Basis of the Frenkel-Band Theory of Association
Equilibrium. J. Chem. Phys..

[99] Peroukidis S. D., Stott I. P., Mavrantzas V. G. (2022). Coarse-Grained
Model Incorporating
Short- and Long-Range Effective Potentials for the Fast Simulation
of Micelle Formation in Solutions of Ionic Surfactants. J. Phys. Chem. B.

[100] Pérez-Sánchez G., Schaeffer N., Greaves T. L., Pereira J. F. B., Coutinho J. A. P. (2022). Tuning the ionic
character of sodium dodecyl sulphate via counter-ion binding: An experimental
and computational study. Front. Mater..

[101] Fontell K. (1981). Liquid Crystallinity in Lipid-Water
Systems. Mol. Cryst. Liq. Cryst..

[102] Patist A., Oh S., Leung R., Shah D. (2001). Kinetics of
micellization: its significance to technological processes. Colloids Surf. A Physicochem. Eng. Asp..

