# Supplementary material for: Coarse-Grained Model of the Sodium Dodecyl Sulfate Anionic Surfactant Based on the MDPD–Martini Force Field
Source: Langmuir. 2026 Mar 4;42(14):9683–92. doi: 10.1021/acs.langmuir.5c05313 (PMC13298907; doi:10.1021/acs.langmuir.5c05313)
Supplement: Supplementary file 1 [file la5c05313_si_001.pdf]

Supporting Information

Coarse-Grained Model of Sodium Dodecyl Sulfate Anionic Surfactant based on the MDPD-Martini Force-Field

Luís H. Carnevale,* Gabriela Niechwiadowicz, and Panagiotis E. Theodorakis

Institute of Physics, Polish Academy of Sciences, Al. Lotników 32/46, 02-668 Warsaw, Poland

E-mail: carnevale@ifpan.edu.pl

Table of contents

Energy equilibration S–2

Micelle Aggregation S–4

Surface Tension Convergence S–5

Concentration Table S–7

Computational time S–8

Energy equilibration

To make sure our systems have reached a stable configuration we plot the change in total energy for all the bulk simulations at different concentrations. All plots were done for the first 10^6 time steps, which is equivalent to 20 ns for MD simulations and 400 ns for MDPD simulations. The dashed blue line represents a rolling average with a window of 100 points.

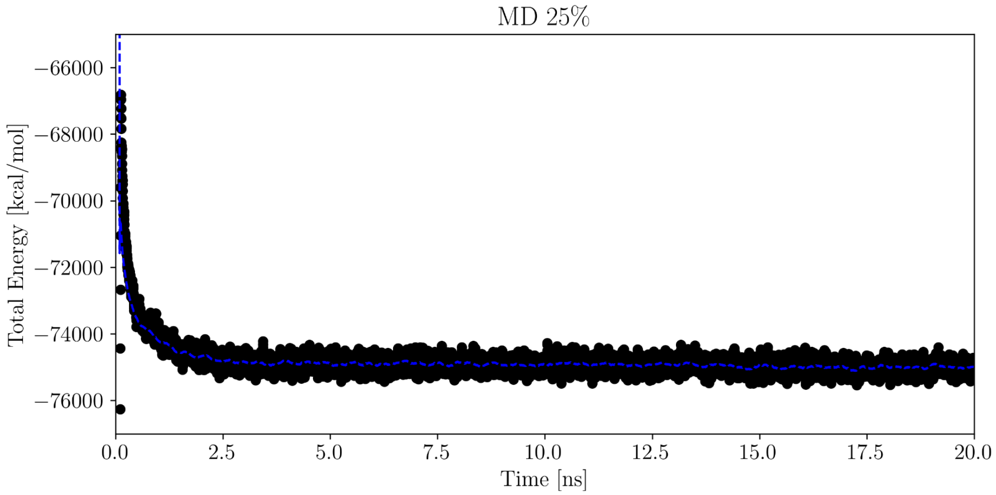

Figure S1: Energy equilibration for the MD bulk system at 25% SDS concentration.

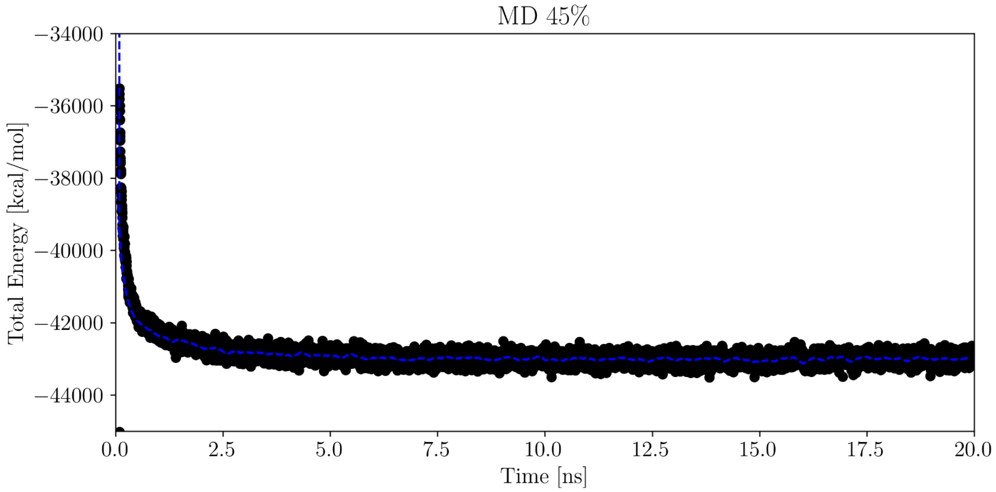

Figure S2: Energy equilibration for the MD bulk system at 45% SDS concentration.

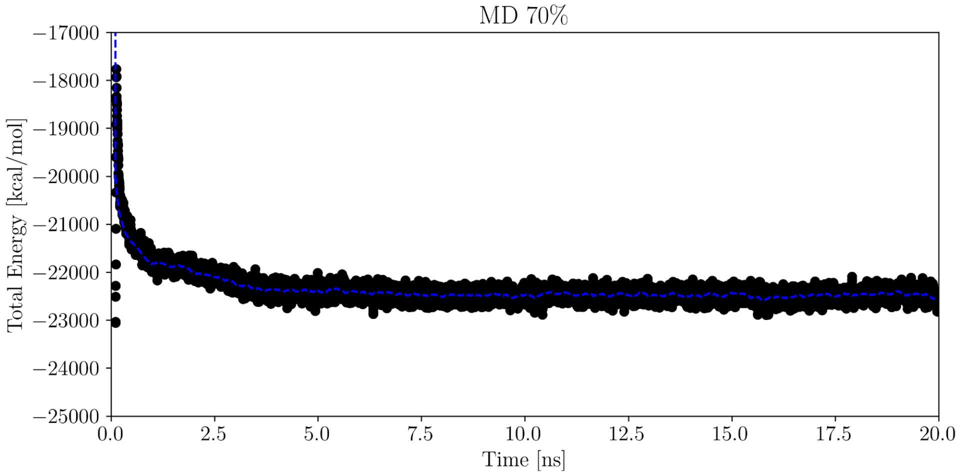

Figure S3: Energy equilibration for the MD bulk system at 70% SDS concentration.

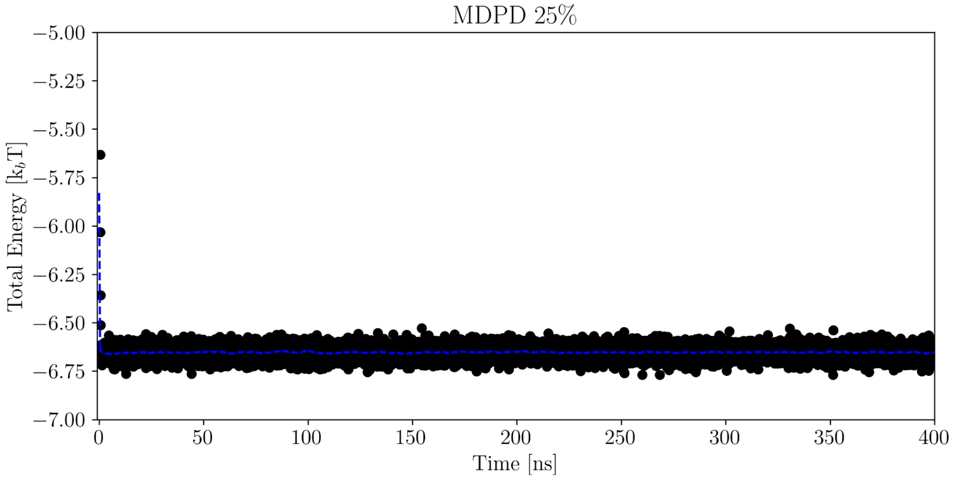

Figure S4: Energy equilibration for the MDPD bulk system at 25% SDS concentration.

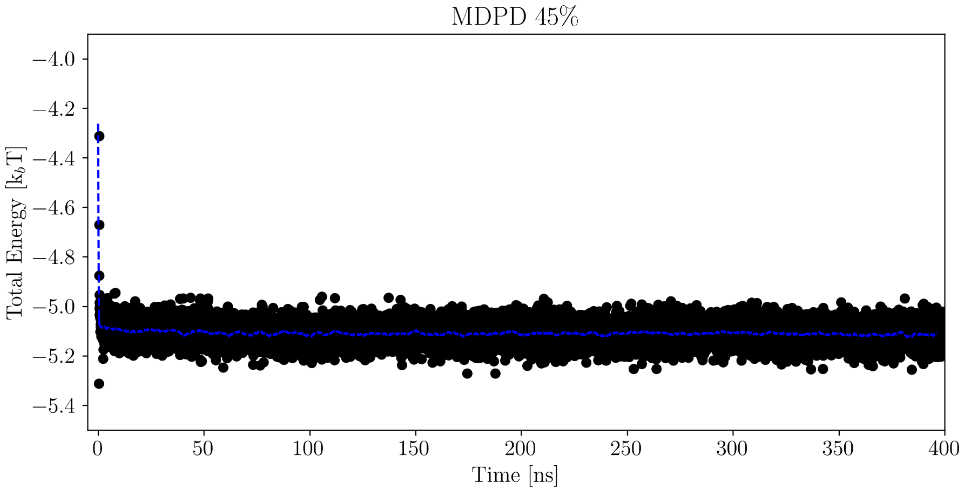

Figure S5: Energy equilibration for the MDPD bulk system at 45% SDS concentration.

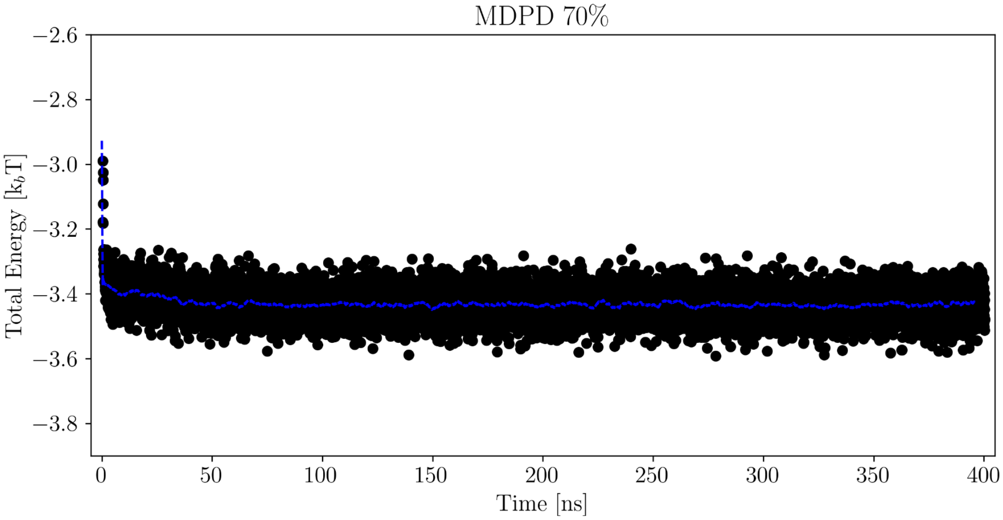

Figure S6: Energy equilibration for the MDPD bulk system at 70% SDS concentration.

Micelle Aggregation

We verified the micelle aggregation convergence not only through the energy equilibration plots, but also by looking at the number of SDS clusters in the simulations. Below is a plot of how the number of SDS clusters change over time. The aggregation number measurements were done when the number of clusters stabilized. From the plot we see that both MD and MDPD aggregation occur similarly and at the same time scale. A histogram presenting the micelle aggregation number distribution used to compute the average value reported in the main text is also shown below.

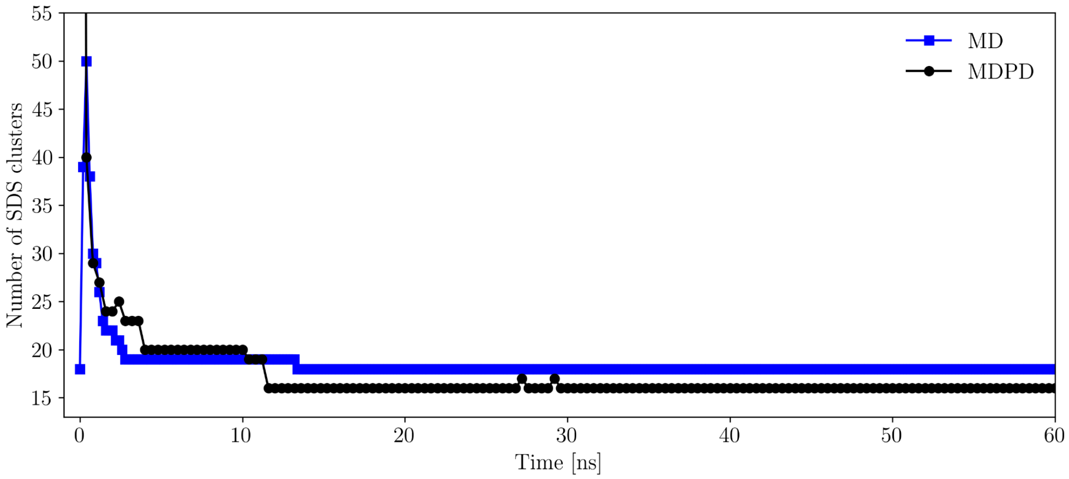

Figure S7: Time evolution of the number of micelles in a single MD and MDPD simulation. Their size distribution was calculated after the plateau level was reached.

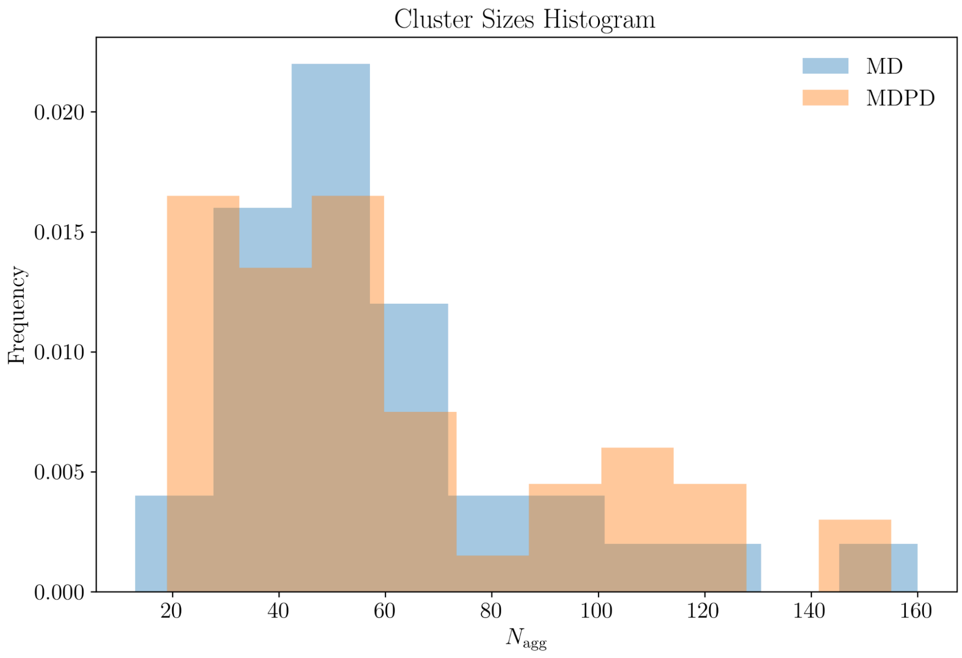

Figure S8: Histogram of the aggregation number distribution for the micelles formed in both MD and MDPD simulations.

Surface Tension Convergence

Below are plots for the surface tension measurements over time for a few surface coverage concentrations. The surface coverage concentration is defined as the number of SDS molecules

divided by the total interfacial area in the system. Both MD and MDPD were sampled over 10^6 time steps after equilibrium was reached. The final results in Figure 2b in the main text were obtained by averaging over time. Energy plots are also shown for the first 20 ns on both MD and MDPD.

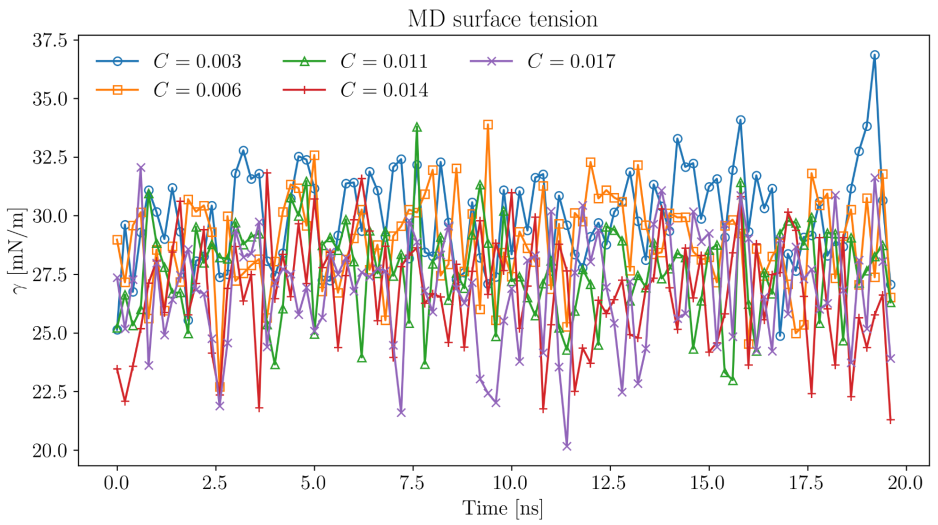

Figure S9: Surface tension measurements over the production run time for MD simulations at different surface coverage concentrations.

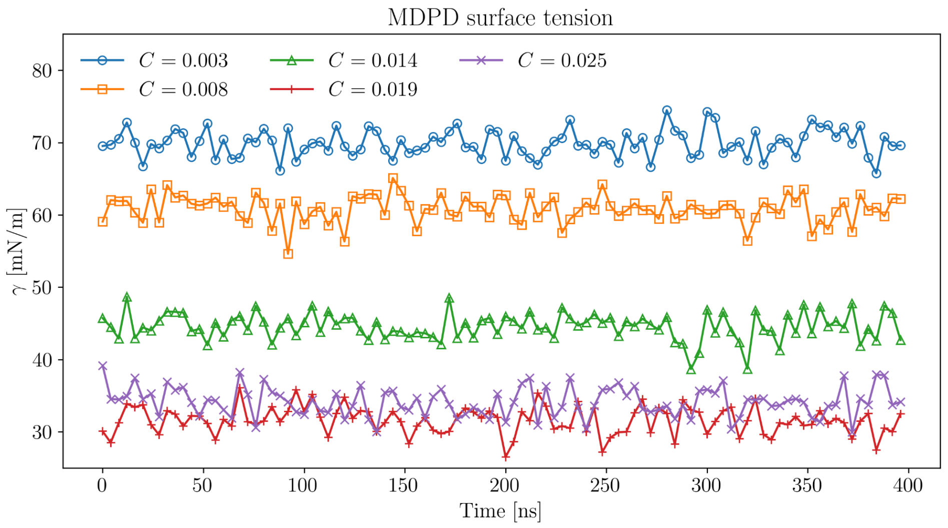

Figure S10: Surface tension measurements over the production run time for MDPD simulations at different surface coverage concentrations.

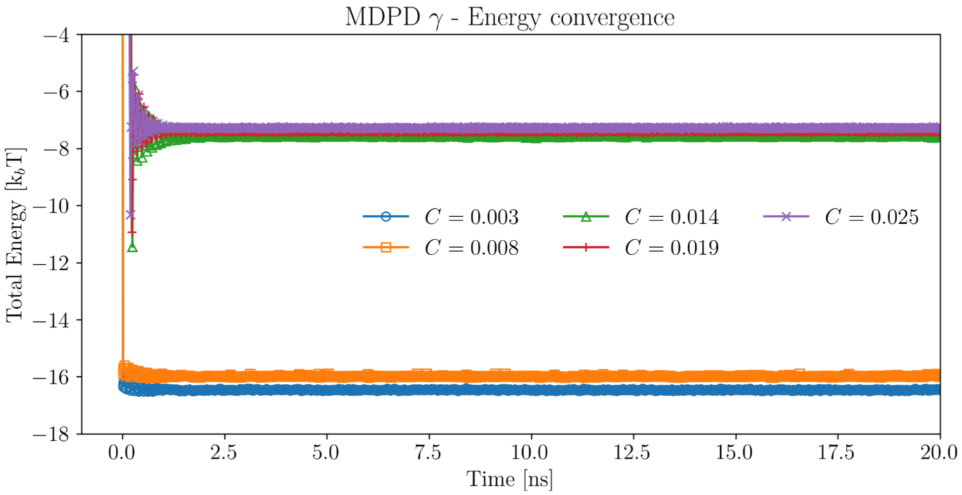

Figure S11: Energy equilibration for a set of slab simulations used to measure surface tension with MDPD.

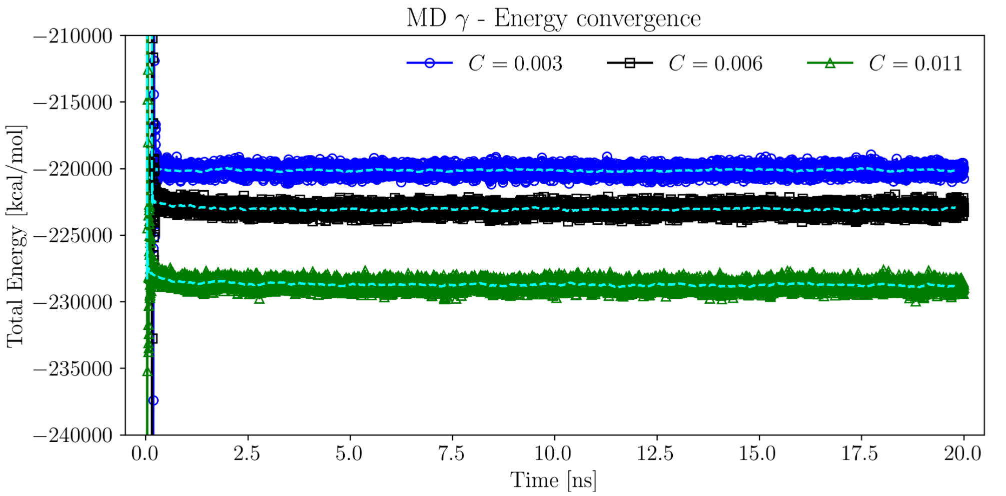

Figure S12: Surface tension measurements over the production run time for MD simulations at different surface coverage concentrations.

Concentration Table

Table with number of beads and molecules for each concentration and simulation conditions. Because the coarse-graining level of $N_m = 3$ used in the MDPD parametrization, more water beads were necessary to compensate the MD-Martini $N_m = 4$. The ratio between MDPD and MD water beads should be $4/3$. The coarse-graining level in our MDPD force-field can and will be adjusted in its future version to match the MD-Martini.

Table S1: Number of molecules and beads for the different concentrations used in the bulk SDS–water simulations.

	Concentration [%]	Number of: SDS	Na	W	Temperature [K]	Pressure [atm]	Time steps
MD	5	1000	1000	75000	300	1	3.00E+06
	25	1000	1000	11000	300	1	3.00E+06
	45	1000	1000	3889	300	1	3.00E+06
	70	1000	1000	714	300	1	3.00E+06
MDPD	5	1000	1000	100000	300	1	3.00E+06
	25	1000	1000	14666	300	1	3.00E+06
	45	1000	1000	5185	300	1	3.00E+06
	70	1000	1000	952	300	1	3.00E+06

Computational time

To compare the computational speed-up in using MDPD, we ran a set of simulations with systems of different sizes and measured the average computational wall time per iteration under the same conditions. All simulations were run on eight cores using an Intel Core i7-10700 @ 2.9Ghz CPU computer. Both MD and MDPD were run with LAMMPS version 29Aug2024. Because of the extra computational cost in calculating the long-range electrostatic interactions, scenarios with them turned on and off were also compared. The results are presented in Table S2 in terms of computational wall time per iteration, and we can see that MDPD actually has a higher computational cost per time-step. However, once we adjust the wall time by the coarse-grained time scaling, MDPD presents a noticeable speed-up in terms of the real time simulated per computational time cost. On average MDPD was four times faster than MD when long-range electrostatics were considered and 15 times faster when no long-range interactions were considered.

Table S2: Computational time comparison between MD and MDPD on eight cores using an Intel Core i7-10700 @ 2.9Ghz CPU.

				With long-range electrostatics		Without long-range electrostatics	
	Number of: SDS	Na	W	Wall Time [ms/iter]	Real Time / Wall time [fs/ms]	Wall Time [ms/iter]	Real Time / Wall time [fs/ms]
MD	100	100	1000	0.567	35.27	0.442	45.25
	200	200	2000	0.963	20.77	0.808	24.75
	1000	1000	10000	4.812	4.16	4.065	4.92
MDPD	100	100	1000	2.943	146.11	0.641	670.8
	200	200	2000	5.298	81.13	1.258	341.81
	1000	1000	10000	25.211	17.06	5.368	80.1
